# Efficacy of *Bidens pilosa* Extract against Herpes Simplex Virus Infection *In Vitro* and *In Vivo*

**DOI:** 10.1155/2012/413453

**Published:** 2012-02-28

**Authors:** Shinji Nakama, Kazumi Tamaki, Chie Ishikawa, Masayuki Tadano, Naoki Mori

**Affiliations:** ^1^Department of Microbiology and Oncology, Graduate School of Medicine, University of the Ryukyus, 207 Uehara, Nishihara, Okinawa 903-0215, Japan; ^2^Musashino Research Institute for Immunity, 790 Nishizatozoe, Gusukube, Miyako Island, Okinawa 906-0106, Japan; ^3^Transdisciplinary Research Organization for Subtropics and Island Studies, University of the Ryukyus, 1 Senbaru, Nishihara, Okinawa 903-0213, Japan

## Abstract

The development of strains of herpes simplex virus (HSV) resistant to drugs has been reported among the immunocompromised patients. Thus, there is a need to develop new therapeutic agents for HSV infections. We evaluated the anti-HSV activity of *Bidens pilosa* (*B. pilosa*), a tropical weed, in tissue culture cells and a mouse model. *B. pilosa* extract showed potent virucidal activity. It inhibited plaque formation and suppressed virus yield in Vero and RAW 264.7 cells infected with HSV-1 and HSV-2. Both the binding of virus to host cells and penetration of virus into cells were also blocked by *B. pilosa*. Furthermore, *B. pilosa* was effective against thymidine kinase-deficient and phosphonoacetate-resistant HSV-1 strains. *B. pilosa* treatment increased the survival rate of HSV-infected mice and limited the development of skin lesions. Our results indicate that *B. pilosa* has anti-HSV activity and is thus a potentially useful medical plant for treatment of HSV infection.

## 1. Introduction

Herpes simplex virus types 1 and 2 (HSV-1 and HSV-2) cause infections worldwide with an estimated 60–95% of adults infected at least by one of them [1]. HSV is transmitted through direct contact of the infected secretions and results in common biological features of herpes viruses, including latency and reactivation [2]. HSV causes a variety of illnesses ranging from asymptomatic infection to fulminant disseminated diseases, such as labial herpes, keratitis, genital herpes, and encephalitis. Several forms of disseminated HSV infection are encountered in patients with reduced cell-mediated immunity such as bone marrow transplant recipients and patients with acquired immunodeficiency syndrome. Acyclovir (ACV) and other nucleoside derivatives have been approved for treatment of HSV infections [1]. However, the increasing clinical application of this type of antiviral agents has resulted in the emergence of drug-resistant herpesvirus strains [3, 4]. In addition, the lack of an effective vaccine and the moderate-to-high toxicity of the available synthetic antiherpes compounds emphasize the need to discover and develop new alternative agents with a different mode of action for the management of HSV infection.

Herbs are potential sources of useful medicinal compounds. *Bidens pilosa Linn. var. radiata *is a tropical weed widely present in the tropical and subtropical regions. This plant was originally found in tropical America and later introduced into the Pacific region and parts of Asia including Miyako Island, Okinawa, Japan. The whole plant or its aerial parts is used in various folk medicines and as a popular ingredient in herbal tea for its anti-inflammatory, antiseptic, liver-protective, blood-pressure lowering, and hypoglycemic effects [5–11]. Although diverse bioactivities have been identified in *B. pilosa*, its antiviral activity has not attracted attention so far. In an effort to discover novel antiviral agents, *B. pilosa* was investigated for its anti-HSV activities in tissue culture cells and in a mouse model.

## 2. Materials and Methods

### 2.1. Reagents

*B. pilosa Linn. var. radiata* was grown from seeds and harvested from January to July on Miyako Island, Okinawa Prefecture, Japan. The harvested aerial parts were washed twice with water, cut into pieces, and then steamed. One hundred kilograms of dried *B. pilosa* were boiled in 1800 litre of water for 2 h. The suspension obtained was treated with the enzyme cellulosine, filtered and concentrated. Finally, *B. pilosa* extract was dried by spraydrier giving 40 kg of a brownish powder. This dried powder was provided by Musashino Research Institute for Immunity Co., Ltd. (Okinawa, Japan). ACV and rutin were purchased from Sigma-Aldrich (St Louis, MO, USA). Phosphonoacetate and caffeic acid were from Wako Pure Chemical Industries (Osaka, Japan). *N* ^G^-nitro-L-arginine methyl ester hydrochloride (L-NAME) was from Dojindo Laboratories (Kumamoto, Japan).

### 2.2. Cells and Viruses

African green monkey kidney Vero cells and murine macrophages transformed with the Abelson leukemia virus RAW 264.7 were propagated in Eagle's Minimum Essential Medium and Dulbecco's modified Eagle's Medium supplemented with antibiotics and 10% fetal bovine serum, respectively. The cells were grown at 37°C in a humidified atmosphere with 5% CO_2_.

 HSV-1 strains HF [12] and 7401H [13], and HSV-2 strain Savage were used. 7401H-derived thymidine kinase-deficient (TK^−^) HSV-1 [14] and phosphonoacetate-resistant (AP^r^) HSV-1 [15] were also used. The viruses were propagated in Vero cells. The virus titer was determined by the plaque assay and expressed as plaque-forming units (PFUs) per mL; virus stocks were stored at −80°C until use.

### 2.3. Cytotoxicity Assay

Vero cells and RAW 264.7 cells were seeded at 1 × 10^4^  cells per well in 96-well plates and grown at 37°C for 24 h. Thereafter, the cells were incubated with various concentrations of *B. pilosa* extract for 72 h at 37°C. Cellular viability was assayed by measuring the mitochondrial-dependent conversion of the water-soluble tetrazolium (WST)-8 (Nacalai Tesque, Kyoto, Japan) to a colored formazan product.

### 2.4. Virucidal Assay

 The direct effects of *B. pilosa* extract, caffeic acid, and rutin on infection of HSV-1 strain HF and HSV-2 strain Savage were evaluated. The concentrated virus suspension (1.2 × 10^6^ PFU/mL) was incubated with an equal volume of Eagle's Minimum Essential Medium containing different concentrations of *B. pilosa* extract (250, 500, 1000, and 2000 *μ*g/mL) at 37°C for 1 h. The infectious virus remaining in the samples was diluted by 100-fold. This dilution essentially eliminated the potential effect of the remaining extract on subsequent binding events. One hundred microliters of the mixed suspensions were then added to subconfluent monolayers of Vero cells at 4°C. After 1 h incubation for virus adsorption, the cell monolayers were washed carefully twice with phosphate buffered saline (PBS), and the residual virus infectivity was determined by plaque assay following the procedures described below. The effect of *B. pilosa* extract on virus infectivity was calculated.

### 2.5. Antiviral Assay Using Plaque Reduction Assay

 The antiviral activity of *B. pilosa* against HSV-1 and HSV-2 was evaluated by the plaque reduction assay. Vero cells were seeded onto 6-well culture plates at a density of 3 × 10^5^ cells per mL and incubated for 24 h. The medium was then discarded and the cell monolayer was infected with a virus suspension to yield 100 plaques per well in the presence of various concentrations of *B. pilosa* extract (125, 250, 500, 1000, and 2000 *μ*g/mL), ACV (3.1, 6.3, 12.5, 25, and 50 *μ*g/mL), or phosphonoacetate (6.3, 12.5, 25, 50, and 100 *μ*g/mL). The virus was allowed to be adsorbed for 1 h at 4°C, then the cells were washed twice with PBS and overlaid with an overlay medium containing 0.5% of methylcellulose and dilutions of *B. pilosa* extract. The plate was further incubated for a time period corresponding to four cycles of multiplication of HSV (72 h) [16]. Later, the overlay medium was removed, and the infected cell monolayer was fixed with 100% methanol. The virus plaques formed on Vero cells were stained with 0.2% crystal violet in 50% methanol.

In order to determine the mode of antiviral action of *B. pilosa* extract, various concentrations of the extract were added at different periods of infection. Confluent monolayers were incubated with plain or extract-containing medium for 1 h before viral challenge at 37°C (effect of cell pretreatment), during viral adsorption at 4°C for 1 h (effect on adsorption), for 1 h at 37°C to allow viral internalization into cells (effect on penetration), and during cultivation at 37°C (effect on replication). After 72 h, the number of plaques of extract-treated cells was compared with untreated controls. All untreated controls were incubated with culture medium instead of the extract.

### 2.6. Virus Yield Reduction Assay

 Vero and RAW 264.7 cell monolayers were infected with the HSV-1-HF and HSV-2-Savage strains. After 1 h of adsorption at 4°C, they were washed twice with PBS and incubated at 37°C in a medium containing 1000 *μ*g/mL *B. pilosa* extract. Twenty-four to 72 h postinfection, the medium was harvested into tubes. The infected cell monolayers were washed twice with PBS and subjected to three cycle of freeze-thawing. Virus yields in the medium and cells were determined separately by the plaque assay.

### 2.7. Reverse Transcriptase-PCR (RT-PCR)

 Total cellular RNA from cells was extracted with Trizol (Invitrogen, Carlsbad, CA, USA) according to the protocol provided by the manufacturer. First-strand cDNA was synthesized from 1 *μ*g total cellular RNA using an RNA-PCR kit (Takara Bio Inc., Otsu, Japan) with random primers. The primers used were 5′-TCATTGTACTCTGAGGGCTGACACA-3′ (forward) and 5′-GCCTTCAACACCAAGGTTGTCTGCA-3′ (reverse) for murine inducible NO synthase (iNOS), and 5′-GTGGGGCGCCCCAGGCACCA-3′ (forward) and 5′-CTCCTTAATGTCACGCACGATTTC-3′ (reverse) for *β*-actin. The length of RT-PCR was 25 cycles for iNOS and 28 cycles for *β*-actin. The PCR products were fractionated on 2% agarose gels and visualized by ethidium bromide staining.

### 2.8. Measurement of Nitric Oxide (NO)

 Nitrite, the stable end product of NO, was measured by the colorimetric assay. Briefly, the medium was removed from individual wells and treated with Griess reagent (1% sulphanilamide and 0.1% naphtylethylene diamine dihydrochloride in 2% H_3_PO_4_) for 10 min at room temperature. The optical density of the samples was obtained using an automated microplate reader at 550 nm. A standard curve using a standard solution of NaNO_2_ in culture medium was employed to calculate the nitrite concentration.

### 2.9. Efficacy of *B. pilosa* Extract in a Cutaneous Mouse HSV-1 Infection Model

 The right mid-flank of each C57Bl/6 mouse was chipped and depilated with a chemical depilatory hair remover. The mice were randomly divided into four groups each comprising 6 mice. Mice of the first group, the control group, were infected but received no treatment. Those of the second group, the extract-treatment group, were infected and treated with the *B. pilosa* extract (see below). The third group was the extract-pretreatment group. An initial oral dose of the *B. pilosa* extract was administered at day 7 before infection, followed by continuous administration of the extract (see below). The final fourth group was the ACV-treatment group. In this group, mice were infected and then treated with ACV. To induce infection, the naked skin was scratched using a 26-guage needle, and 10 *μ*L of HSV-1 (7401H strain) suspension of 1 × 10^6^ PFU was applied to the scratched area, as described previously [13]. *B. pilosa* extract (1 g/kg per dose) or ACV (5 mg/kg per dose) was administered orally by gavage twice daily at 1 h after HSV-1 inoculation and for 18 successive days after viral infection. Oral administration of *B. pilosa* also commenced at day 7 prior to viral inoculation and continued thereafter for 18 days in the extract-pretreatment group.

The appearance of skin lesions was continuously observed daily and scored as follows: 0, no lesion; 2, vesicles in local region; 4, erosion and/or ulceration in the local region; 6, mild zosteriform lesion; 8, moderate zosteriform lesion; 9, severe zosteriform; 10, death. The infected mice were held at least for 18 days after infection. The toxicity of *B. pilosa* extract was assessed in the treated mice by the loss of body weight compared with the control group. The mice were weighed once a week. This experiment was performed according to the Guidelines for Animal Experimentation of the University of the Ryukyus and approved by the Animal Care and Use Committee of the University of the Ryukyus.

## 3. Results

### 3.1. Cytotoxicities of *B. pilosa* in Vero and RAW 264.7 Cells

**Figure 1:**
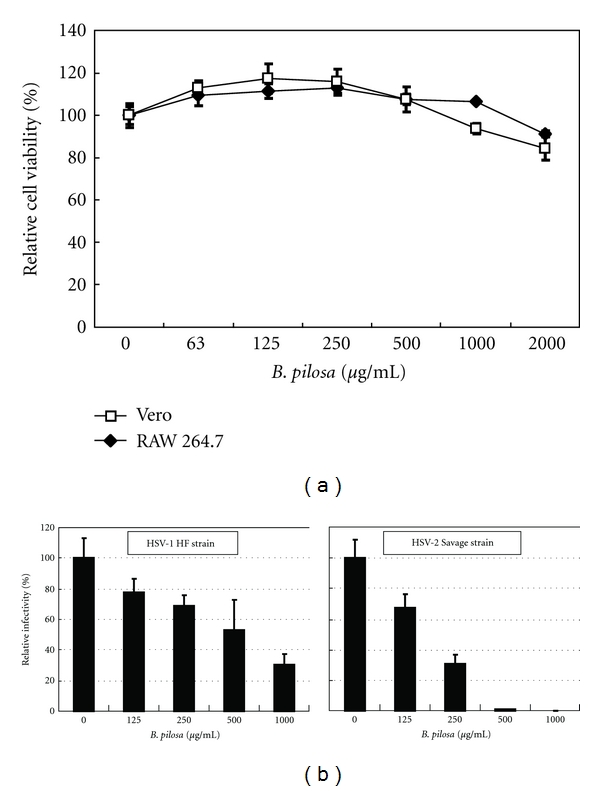
Cytotoxic and virucidal effects of the extract of *B. pilosa*. (a) Cell viability was determined in triplicate cultures by WST-8 assay. The results are expressed as percentage of control and are mean ± SD of triplicate cultures. (b) Effects of the extract of *B. pilosa* on HSV-1 and HSV-2 infectivity. Viruses were incubated for 1 h at 37°C with increasing concentrations of the extract and immediately tested in a plaque reduction assay. Data are presented as % of control. Data are mean ± SD of triplicate cultures.

First, the concentration that did not affect cell viability that could be used in subsequent assays was determined. For this purpose, the WST-8 assay was employed to determine the cytotoxic effect of *B. pilosa* extract in Vero and RAW 264.7 cells (Figure 1(a)). Subconfluent monolayers of Vero cells treated with *B. pilosa* at concentrations ≤ 2000 *μ*g/mL did not show any cytotoxic effects. Moreover, *B. pilosa* was also nontoxic against RAW 264.7 cells at ≤ 2000 *μ*g/mL (Figure 1(a)). Microscopic examination of cell monolayers after 72 h of incubation with *B. pilosa* at 2000 *μ*g/mL showed no changes in cell morphology compared with the control (data not shown). Therefore, *B. pilosa* was used at concentrations ≤ 2000 *μ*g/mL in subsequent studies.

### 3.2. Virucidal Effect of *B. pilosa* Extract on HSV-1 and HSV-2 Infectivity

 The virucidal effect of *B. pilosa* was evaluated by incubating the extract with HSV-1 and HSV-2. As shown in Figure 1(b), *B. pilosa* reduced infectivity of HSV-1 strain HF and HSV-2 strain Savage depending on the dosage level. These results indicate that *B. pilosa* directly inactivated the viruses with potent virucidal effects against HSV-1 and HSV-2.

### 3.3. Virucidal Activity of Selected Constituents

**Figure 2:**
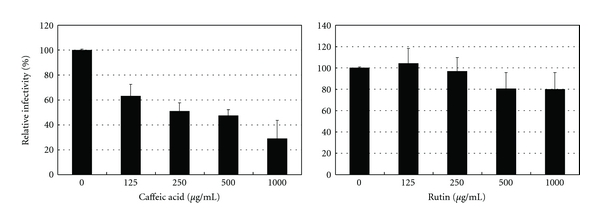
Effect of caffeic acid and rutin on infectivity of the HSV-1 HF strain. Viruses were incubated for 1 h at 37°C with increasing concentrations of caffeic acid and rutin and immediately tested by the plaque reduction assay. Data are presented as % of control. Data are mean ± SD of triplicate cultures.

 Six caffeic acid derivatives (neochlorogenic acid, chlorogenic acid, 4-*O*-caffeoylquinic acid, 3,4-di-*O*-caffeoylquinic acid, 3,5-di-*O*-caffeoylquinic acid, and 4,5-di-*O*-caffeoylquinic acid) and 7 flavonoids (rutin, quercetin, quercetin derivative, hyperin, isoquercitrin, centaurein, and jacein) have been isolated from *B. pilosa* [17]. The potential viral inactivation effect of caffeic acid and rutin was determined against HSV-1. Caffeic acid, but not rutin, exhibited a concentration-dependent virucidal effect (Figure 2).

### 3.4. Effect of *B. pilosa* Extract in Inhibiting Plaque Formation

**Figure 3:**
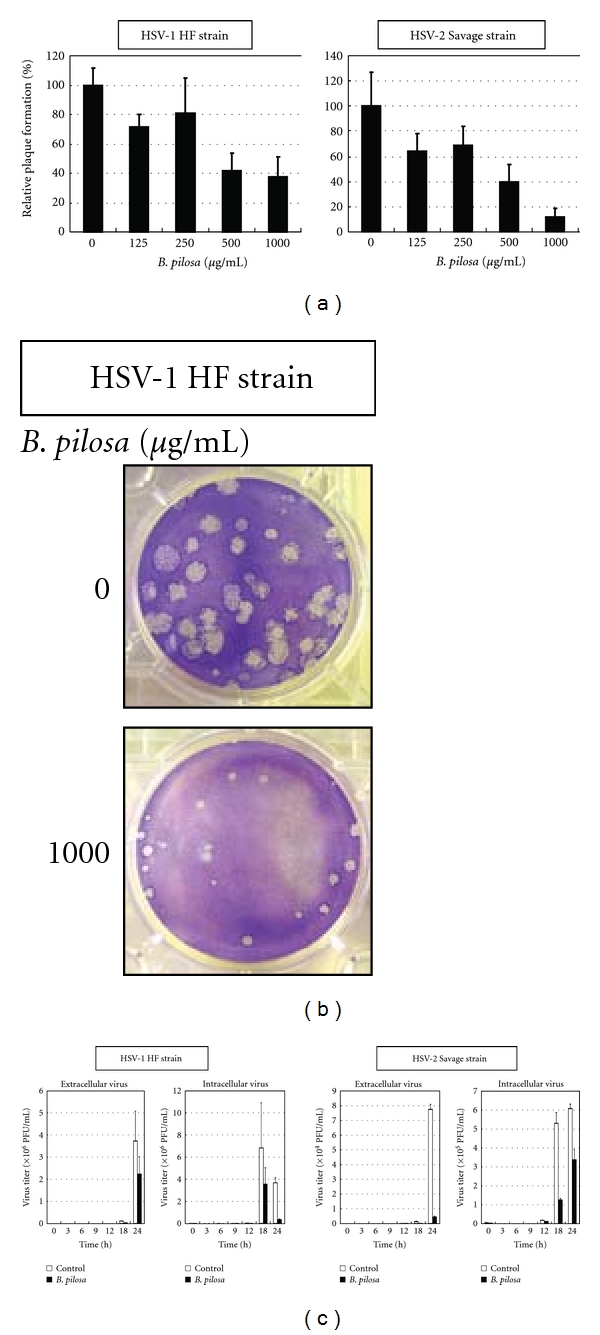
Effects of the extract in inhibition of plaque formation and on reduction of virus yield. (a) Effect of the extract in inhibition of plaque formation. Vero cell monolayers were infected with HSV-1 and HSV-2 in the presence of various concentrations of *B. pilosa* extract. After 1 h incubation for virus adsorption, the prescription was washed out and cells were overlaid with an overlay medium containing the extract for 72 h period of infection. Data are % of control and represent mean ± SD of triplicate cultures. (b) Treatment with 1000 *μ*g/mL *B. pilosa* extract decreased the number and size of plaques. (c) Effect of the extract on reduction of virus yield. Vero cell monolayers were infected with HSV-1 and HSV-2 and left for adsorption for 1 h. They were washed and overlaid with an overlay medium containing 1000 *μ*g/mL of *B. pilosa* extract. At the indicated hours postinfection, both cell-associated and extracellular viruses were titrated by the plaque assay.

 The plaque reduction assay demonstrated that *B. pilosa* extract inhibited plaque formation of HSV-1 and HSV-2, and this effect was dose-dependent (Figure 3(a)). Furthermore, the number and size of plaques decreased in the presence of *B. pilosa* extract (Figure 3(b)). To confirm that *B. pilosa* inhibits virus growth, both cell-associated and extracellular viruses were separately titrated by the plaque assay. HSV-1- and HSV-2-infected cells were treated with *B. pilosa* for 24 h as described in Section 2. Under these conditions, a time-dependent reduction in the yield of both cell-associated and extracellular viruses was observed at 1000 *μ*g/mL of *B. pilosa* (Figure 3(c)).

### 3.5. Activity of *B. pilosa* Extract against Wild-Type and Drug-Resistant HSV-1 Strains

**Figure 4:**
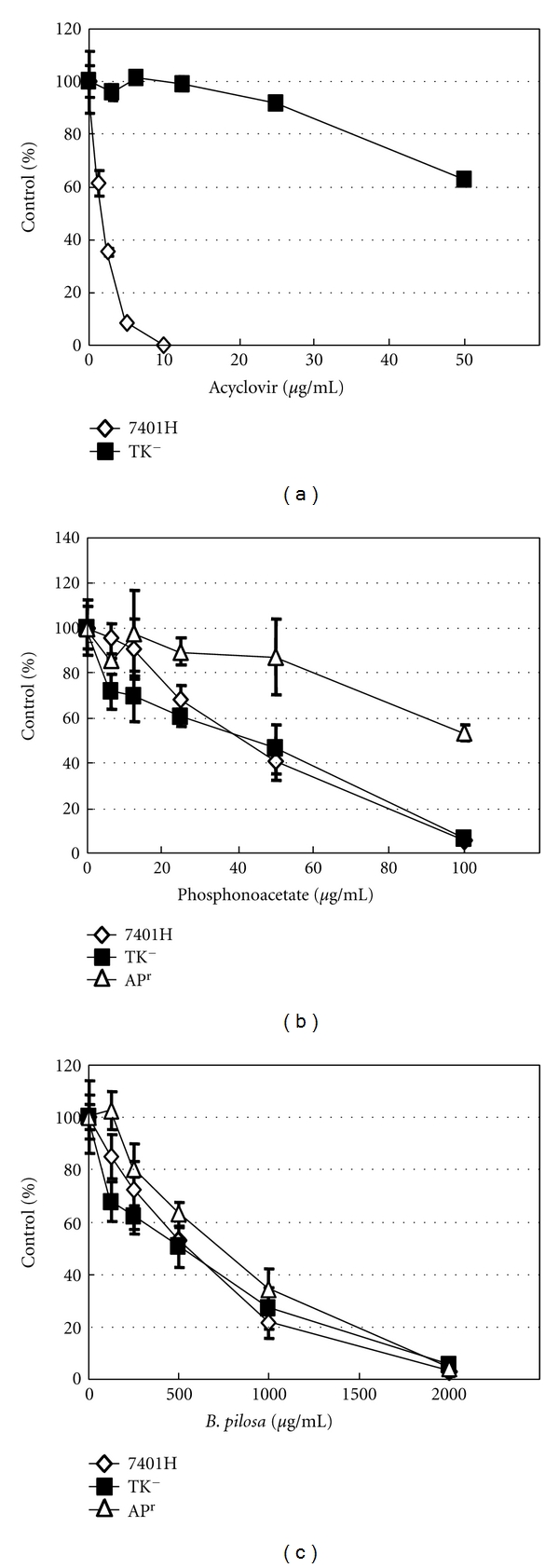
Activity of ACV (a), phosphonoacetate (b), and *B. pilosa* extract (c) on the plaque formation of wild-type and resistant strains of HSV-1 in Vero cells. Data are % of control and represent mean ± SD of triplicate cultures.

 The antiviral activities of *B. pilosa* against HSV-1 TK^−^, AP^r^, and wild-type 7401H strains were examined to evaluate its anti-HSV-1 modes of action. As shown in Figures 4(a) and 4(b), TK^−^ and AP^r^ strains were resistant to ACV and phosphonoacetate, respectively. However, the susceptibility of TK^−^ and AP^r^ strains to *B. pilosa* extract was similar to that of the wild-type strain (Figure 4(c)).

### 3.6. Effects of *B. pilosa* Extract on RAW 264.7 Cells

**Figure 5:**
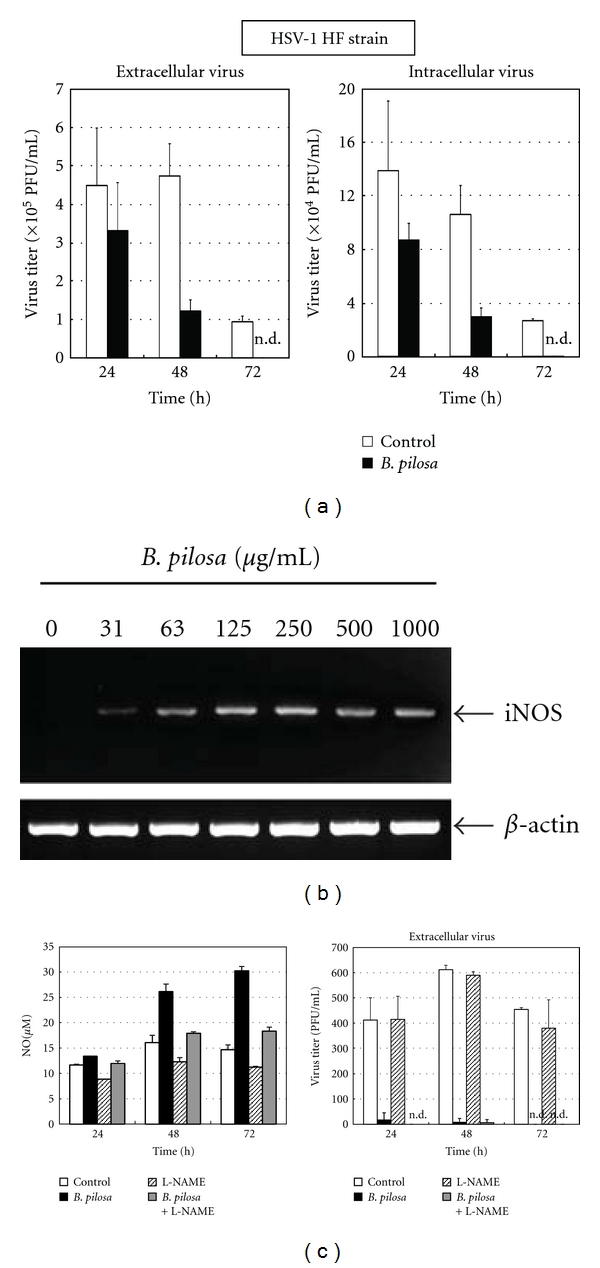
Effects of *B. pilosa* extract on RAW 264.7 cells. (a) Inhibition of HSV-1 replication by *B. pilosa* extract in RAW 264.7 cells. RAW 264.7 cells were infected with HSV-1 and left for adsorption for 1 h. They were washed and then incubated with 1000 *μ*g/mL *B. pilosa* extract. At the indicated hours postinfection, both cell-associated and extracellular viruses were titrated by the plaque assay. Data represent mean ± SD of triplicate cultures. (b) Effect of *B. pilosa* extract on iNOS mRNA expression in RAW 264.7 cells. The cells were incubated with different concentrations of *B. pilosa* extract for 2 h. iNOS mRNA expression was detected by RT-PCR. (c) Effect of L-NAME on HSV-1 replication in RAW cells. Monolayers of RAW 264.7 cells were inoculated with HSV-1 and then treated with 1000 *μ*g/mL *B. pilosa* extract in the presence or absence of 5 mM L-NAME. Cells and supernatants were harvested at 24–72 h after infection for titration of intracellular and extracellular HSV-1 and NO production. Data represent mean ± SD of triplicate cultures. n.d., not detected.

 Although the host defense mechanism is complex, macrophages are key participants in the innate immune system response to the invasion of pathogenic organisms. We therefore investigated the antiviral effects of *B. pilosa* extract in macrophages. Confluent monolayers of RAW 264.7 murine macrophage cells were inoculated with HSV-1, washed out, and then incubated with or without *B. pilosa* extract. After 24–72 h of infection, virus yields in the medium and cells were determined separately by the plaque assay. A time-dependent reduction in the yield of both cell-associated and extracellular viruses was observed at 1000 *μ*g/mL *B. pilosa* (Figure 5(a)).

 Previous work indicated that an NO-mediated antiviral mechanism may be operative in HSV-1-infected RAW 264.7 cells [18]. To establish that iNOS is induced in RAW 264.7 cells after infection, we analyzed the expression of iNOS mRNA. *B. pilosa* extract induced iNOS mRNA expression in a dose-dependent manner (Figure 5(b)). In the next series experiments, an inhibitor of NOS was used to determine if NO contributed to the inhibition of HSV-1 replication in RAW 264.7 cells. After inoculation of RAW 264.7 cells with HSV-1, the monolayers were treated with *B. pilosa* extract in the presence or absence of the competitive inhibitor, L-NAME. As shown in Figure 5(c), *B. pilosa* extract stimulated the production of NO from RAW 264.7 cells in a time-dependent manner. The addition of L-NAME to the medium decreased the production of NO. However, L-NAME failed to reduce the ability of RAW 264.7 cells treated with *B. pilosa* to inhibit HSV-1 replication. These data suggest that NO-mediated antiviral mechanisms do not seem operative in *B. pilosa*-treated macrophages.

### 3.7. Time of Addition Studies

**Figure 6:**
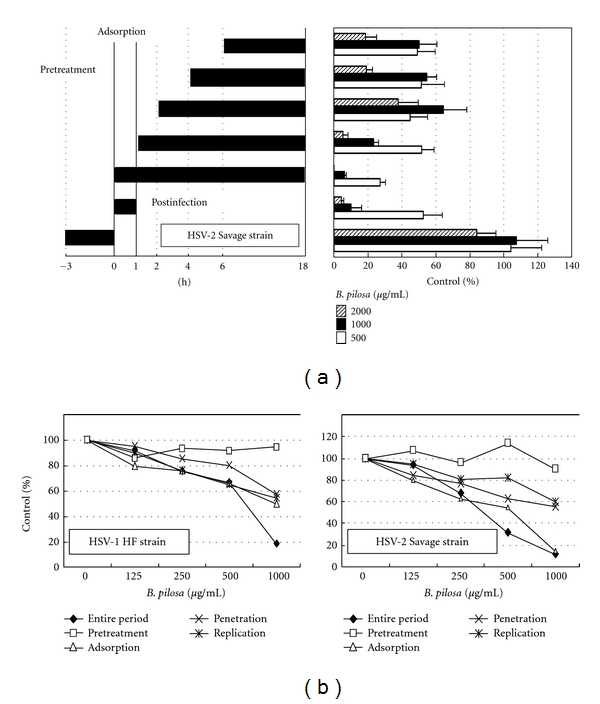
Time of addition studies. (a) Mode of inhibitory effect of *B. pilosa* extract against HSV-2 during different periods of the viral replication cycle. Vero cells were treated with 500, 1000, and 2000 *μ*g/mL *B. pilosa* extract. The extract was added prior to infection (−3 h), during adsorption (0 h), or after infection (1, 2, 4, and 6 h). The prescription was either washed out at indicated times or continuously remained present for the entire 18 h period of infection (left panel). The antiviral activity was determined in Vero cells by the plaque reduction assay. After 18 h, the number of plaques of extract-treated cells was compared with untreated controls. Data are % of control and represent mean ± SD of triplicate cultures. (b) Vero cells were incubated with plain or extract-containing medium for 1 h before HSV-1 challenge (pretreatment), during viral adsorption for 1 h (adsorption), for 1 h to allow viral internalization into cells (penetration), and during cultivation (replication). After 72 h, the number of plaques of extract-treated cells was compared with untreated controls. Data are presented as % of control.

 The inhibitory effect of *B. pilosa* extract was determined following its addition at different time intervals relative to virus infection. The results showed that various concentrations of *B. pilosa* extract added at 3 h prior to virus infection and then being washed out before the infection did not have antiviral activity (Figure 6(a)). In contrast, the concurrent addition of *B. pilosa* extract with virus and washing the extract out at 1 h postinfection inhibited HSV-2 infection. In a single cycle of virus replication, inhibition of virus infectivity was most pronounced when *B. pilosa* was added concurrently with virus infection, with continued presence of the *B. pilosa* extract for the entire 18 h period of infection. The inhibition of plaque formation was observed when *B. pilosa* extract was added at between 1 and 6 h after virus infection. Delaying the time of adding extract failed to significantly decrease its antiviral activity. In other words, the inhibitory effect of *B. pilosa* extract on the virus was persistent even when added 6 h after infection. These results indicate that *B. pilosa* extract affected viral adsorption, penetration, and replication.

 In order to investigate the stage at which the *B. pilosa* affected the viral life cycle, experiments on the time of addition were performed. (1) Monolayers were incubated with the extract for 1 h, washed with PBS, and challenged with HSV (pretreatment). (2) After adsorption for 1 h at 4°C in the presence of extract, the cells were washed with PBS and incubated with culture medium (adsorption). (3) After adsorption, the medium was replaced with extract-containing medium, the cells were washed with PBS, and overlaid with an overlay medium (penetration). (4) After penetration, the cells were washed with PBS, overlaid with an overlay medium containing extract, and further incubated for 72 h (replication). (5) The extract was added at 1 h prior to virus infection and remained present for the entire 72 h period of infection (entire period). The results showed that the extract added at 1 h prior to virus infection and then washed out did not have antiviral activity (Figure 6(b)), similar to the data shown in Figure 6(a). In contrast, adding extract before infection, with continued presence of the extract for the entire 72 h period of infection, resulted in marked reduction of plaque formation, and this effect was dose-dependent. Its presence during viral adsorption, penetration, and replication reduced plaque formation in a dose-dependent manner (Figure 6(b)).

### 3.8. Efficacy of *B. pilosa* Extract in a Cutaneous Mouse HSV-1 Infection Model

**Figure 7:**
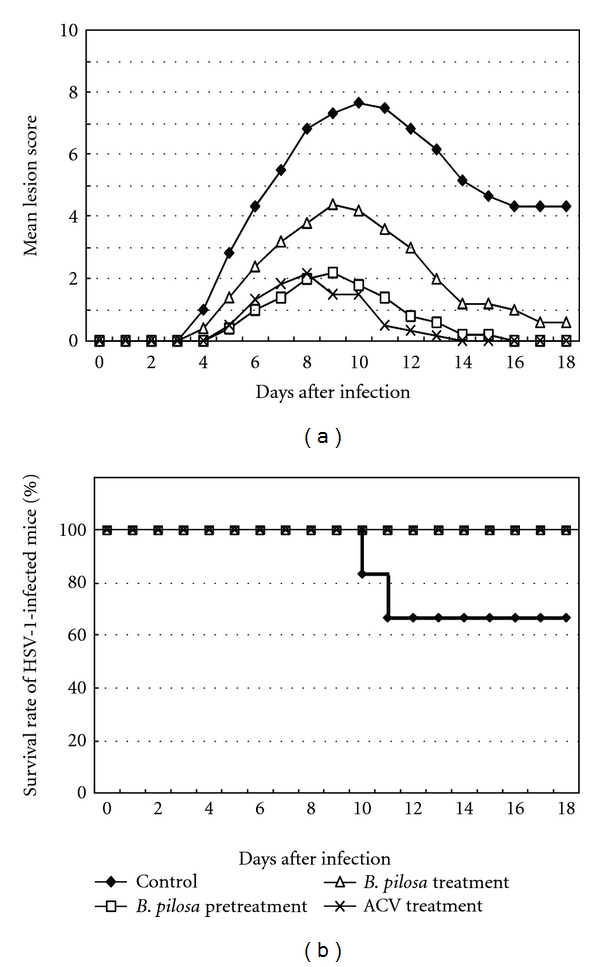
Effects of *B. pilosa* extract on mice cutaneously infected with HSV-1. Six mice in each group were infected. Extract (1 g/kg per dose) and ACV (5 mg/kg per dose) were orally applied twice daily before or after infection. Mice were monitored everyday for development of skin lesions (a) and survival (b). ◆, control (water treatment); □, *B. pilosa* extract pretreatment; ∆, *B. pilosa* treatment; ×, ACV treatment.

 The anti-HSV-1 activity of *B. pilosa* was evaluated in the skin of HSV-1-infected mice. Previously, we found that *B. pilosa* extract was well tolerated and no side effect was observed when four-week-old rats were administered orally with *B. pilosa* extract at 2 g/kg, once a day, for 14 days (data not shown). Therefore, the *B. pilosa* extract was administered orally every 12 h for 18 days after infection at 1 g/kg per dose. Treatment with *B. pilosa* extract delayed the development and progression of skin lesions compared with the control. The anti-HSV-1 efficacy of *B. pilosa* was further analyzed by pretreatment of infected animals. In these experiments, mice received *B. pilosa* twice daily for 7 days before HSV-1 infection, and treatment was continued till 18 days after HSV-1 infection. Pretreatment with *B. pilosa* delayed the development and progression of skin lesions similar to ACV treatment. Two mice of the control group died during the 18-day treatment period, but all mice treated with *B. pilosa* or ACV after HSV-1 infection, and those pretreated with *B. pilosa,* were still alive at 18 days after infection (Figure 7(b)). Toxicity studies of *B. pilosa* extract in infected mice based on frequent measurement of body weights indicated that *B. pilosa* has no toxic effects (data not shown).

## 4. Discussion

Studies had shown that the extracts and several constituents of *B. pilosa* exhibit various clinical effects, whereas only one report has shown that hot water extract of *B. pilosa* had antiviral activity [19]. The present study demonstrated the antiviral activity of *B. pilosa* extract against HSV-1 and HSV-2 both *in vitro* and *in vivo*. *In vitro*, *B. pilosa* extract did not prevent Vero cells from HSV-1 and HSV-2 infection in the pretreatment assay, but showed virucidal effect against HSV-1 and HSV-2. The time of addition experiments suggested multiple targets of activity—*B. pilosa* extract inhibited virus adsorption to cells and affected some intracellular steps of viral replication. Our results imply that *B. pilosa* seems to inhibit HSV biological synthesis and directly inactivate the viruses, and blocking their adsorption to the cells *in vitro*. Furthermore, the virucidal effect of one constituent, caffeic acid, was determined against HSV-1 *in vitro*. This phenomenon may be explained by the nature of plant extracts, which typically contain many compounds with different physicochemical characteristics. The extracts can thus be accepted as being the sum of different pharmacologically active substances, in certain cases even acting antagonistically or synergistically, demonstrating polyvalent action by interfering with different steps of the virus replication cycle.

Our results showed that both the wild and resistant strains of HSV-1 were sensitive to the extract. Since the mechanisms of action of ACV and phosphonoacetate are well understood, it is therefore conceivable that the mechanism of action of the extract against these resistant viral strains is different from that of the antiviral action of ACV and phosphonoacetate. Further investigation of the mechanism of action is needed.

NO is being recognized as an important component of the host response to infection. Since NO has antiviral activity [18] and RAW 264.7 cells can be stimulated to overproduce NO by *B. pilosa* extract, we examined whether NO-mediated antiviral mechanism could explain the inhibitory effects of *B. pilosa* extract on HSV-1 replication in RAW 264.7 cells. Indeed, RAW 264.7 cells were permissive to HSV-1 replication. However, the inhibitor of NOS, L-NAME, did not reduce the ability of RAW 264.7 cells to suppress HSV-1 replication by *B. pilosa* extract. These results suggest that NO does not contribute to the antiviral activity of *B. pilosa* extract.

We employed an HSV-infected C57Bl/6 mouse model suitable for screening and evaluating anti-HSV agents. Cutaneous inoculation of HSV-1 strain resulted in the development of skin lesions in all mice and death of some mice. We used 10 mg/kg/day of ACV and 2 g/kg/day of *B. pilosa* extract for oral administration to mice in this HSV-1 infection model. The mice treated with *B. pilosa* extract before and after infection showed better survival rate compared to the virus control group. Furthermore, *B. pilosa* extract delayed the development and progression of skin lesion. Furthermore, treatment with the extract before infection showed more effective therapeutic anti-HSV-1 efficacy than treatment after infection. The results suggest the beneficial effects of this extract in preventing reactivation of HSV.

The protective effect of *B. pilosa* in mice may be related to an immunomodulatory activity as well as a direct antiviral effect. The extract of *B. pilosa* and two bioactive flavonoids from *B. pilosa* were reported to stimulate the expression of interferon-*γ*, a potent cytokine [20]. Considered together, this plant extract is potentially useful therapeutically against HSV infection.

The utilization of medicinal plant extracts as drugs, medicines, or supplements based on their biological activities examined scientifically allows their use in health care services. Therefore, *B. pilosa* extract could be clinically useful when used prophylactically or therapeutically as an anti-HSV medicine and could be effective against ACV-resistant variants encountered during ACV therapy. The mechanism of action and clinical study of this extract need further investigations.

## 5. Conclusions

Hot water extract of *B. pilosa* showed activity against HSV-1 and HSV-1 both *in vitro* and *in vivo*. The present study has demonstrated the potential clinical application of *B. pilosa*.
